# Supplementary material for: Electric field manipulation enhanced by strong spin-orbit coupling: promoting rare-earth ions as qubits
Source: Natl Sci Rev. 2020 Jun 27;7(10):1557–63. doi: 10.1093/nsr/nwaa148 (PMC8288692; doi:10.1093/nsr/nwaa148)
Supplement: nwaa148_Supplemental_File [file nwaa148_supplemental_file.docx]

## Supporting Information

# Electric field manipulation enhanced by strong spin-orbit coupling: promoting rare earth ions as qubits

Zheng Liu^1^, Ye-Xin Wang^1^, Yu-Hui Fang^1^, Si-Xue Qin^2^, Zhe-Ming Wang^1^, Shang-Da Jiang^1,3*^, and Song Gao^1,3,4*^

1. Beijing National Laboratory of Molecular Science, State Key Laboratory of Rare Earth Materials Chemistry and Applications, Beijing Key Laboratory of Magnetoelectric Materials and Devices, College of Chemistry and Molecular Engineering, Peking University, Beijing 100871, P. R. China. E-mail: jiangsd@pku.edu.cn; gaosong@pku.edu.cn
2. Department of Physics, Chongqing University, Chongqing 401331, P. R. China
3. Beijing Academy of Quantum Information Sciences, West Bld.#3, No.10 XiBeiWang East Rd., HaiDian District, Beijing 100193, P. R. China
4. School of Chemistry and Chemical Engineering, South China University of Technology, Guangzhou 510640, P. R. China

**S1 Crystal Information**

**S2 Electric-Spin Coupling Hamiltonian**

**S3 Echo Decay and Recovery**

### S1 Crystal Information

YAG crystalizes in a cubic unit cell of Ia-3d with a = 12.88Å. Ce^3+^ cations are doped into YAG crystal occupying the Y^3+^ positions. There are 36 sites of Y^3+^ and eight inversion centers, (1/2,1/2,1/2) and (1/2±1/4,1/2±1/4,1/2±1/4) in one unit cell (Figure S1-1).


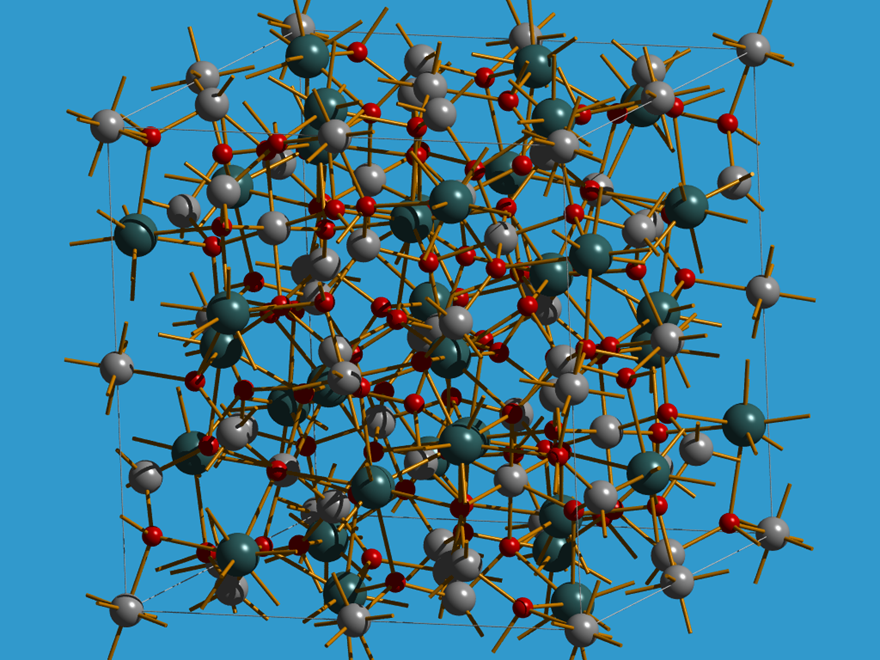


**Figure S1-1** The unit cell of Ce:YAG single crystal.

The 36 Y^3+^ sites can be divided into 6 magnetic inequivalent groups, marked as Ce-1 to Ce-6. Each Ce^3+^ ion is surrounded by 8 oxygen nuclei with the local symmetry of D_2_. The three C_2_ axes form the magnetic principal axes, whose vectors are shown in Table S1-2 in terms of the crystal coordinates.

**Table S1-2** Magnetic principal axes of each Ce^3+^ ions

| principal axes | Ce-1 | Ce-2 | Ce-3 | Ce-4 | Ce-5 | Ce-6 |
| --- | --- | --- | --- | --- | --- | --- |
| ***x*** axis | 1, 0, 1 | 0, 1, 1 | 1, -1, 0 | 1, 0, -1 | 0, 1, -1 | 1, 1, 0 |
| ***y*** axis | 1, 0, -1 | 0, 1, -1 | 1, 1, 0 | 1, 0, 1 | 0, 1, 1 | 1, -1, 0 |
| ***z*** axis | 0, 1, 0 | -1, 0, 0 | 0, 0, 1 | 0, -1, 0 | 1, 0, 0 | 0, 0, -1 |

The applied electric field is normal to the crystal face (111). The local electric field for the 6 groups of Ce^3+^ ions are shown in Table S1-3

**Table S1-3** The electric field strength for each Ce^3+^.

|  | ***E_x_*** | ***E_y_*** | ***E_z_*** |
| --- | --- | --- | --- |
| Ce-1 | $\sqrt{6}/3E$ | $0$ | $\sqrt{3}/3E$ |
| Ce-2 | $\sqrt{6}/3E$ | $0$ | $\sqrt{3}/3E$ |
| Ce-3 | $0$ | $\sqrt{6}/3E$ | $\sqrt{3}/3E$ |
| Ce-4 | $0$ | $\sqrt{6}/3E$ | $\sqrt{3}/3E$ |
| Ce-5 | $0$ | $\sqrt{6}/3E$ | $\sqrt{3}/3E$ |
| Ce-6 | $\sqrt{6}/3E$ | $0$ | $\sqrt{3}/3E$ |


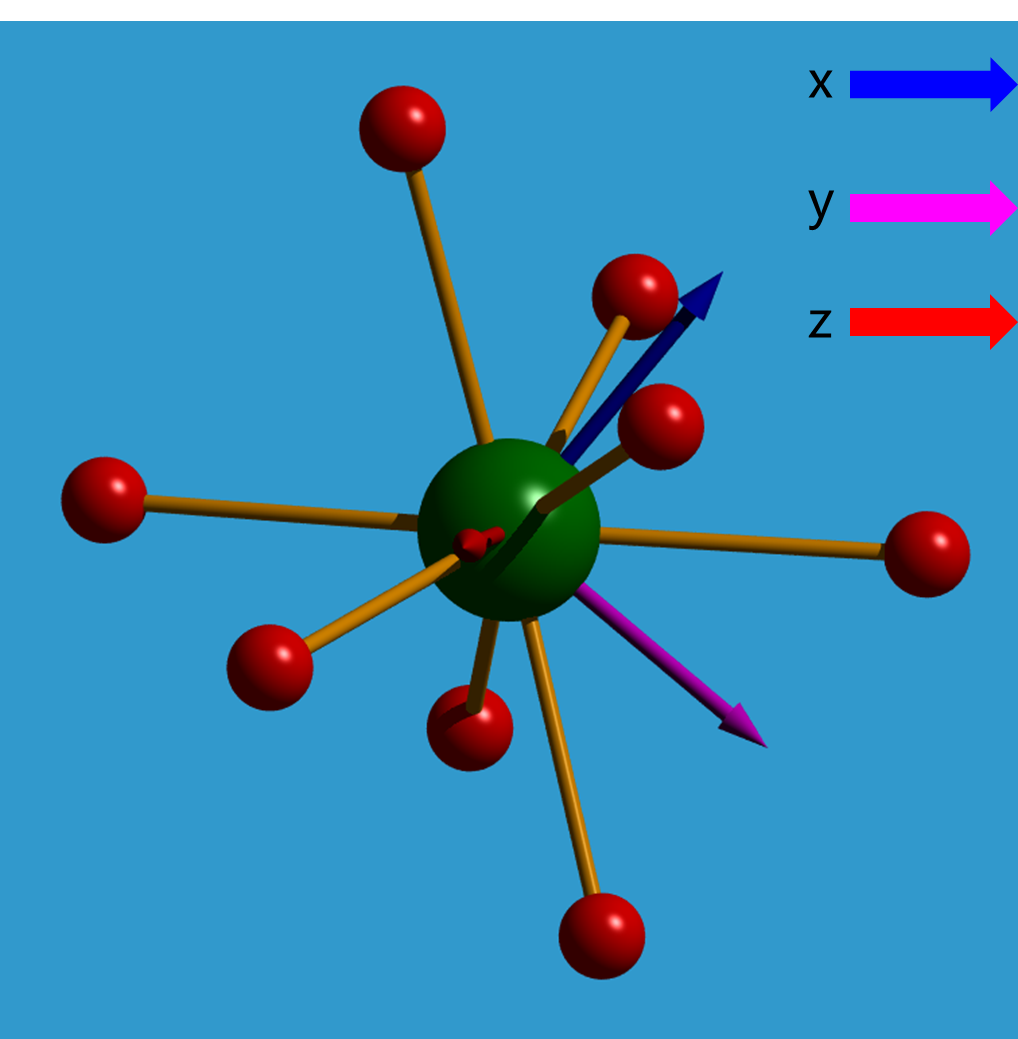


**Figure S1-4** Local environment of Ce^3+^ and its magnetic principle axes.


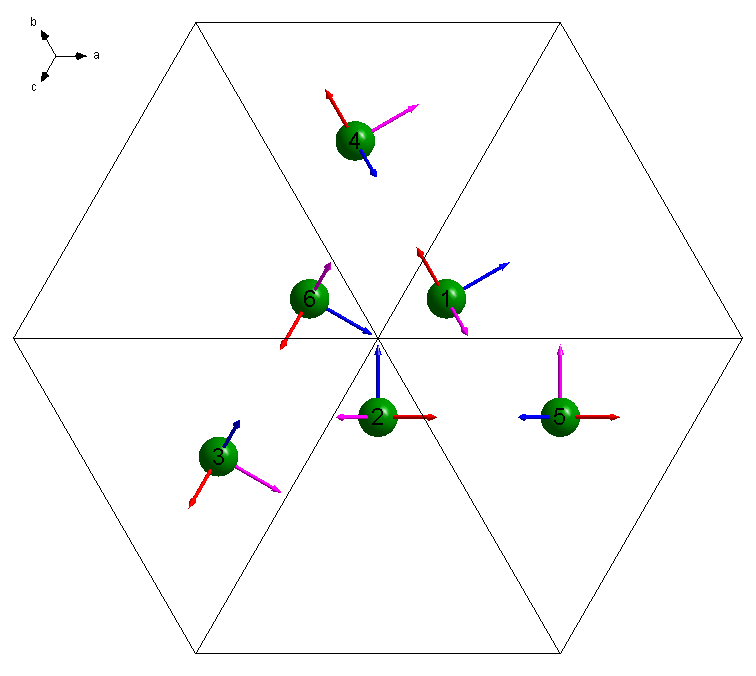


**Figure S1-5** The electric field strength for each Ce^3+^. The external electric field is perpendicular to the paper.

### S2 Electric-Spin Coupling Hamiltonian

The electric-spin coupling Hamiltonian can be written as

$$\begin{aligned} \hat{H}_{E}=\sum_{ijk} E_{i}T_{ijk}\mu_{B}B_{j}\hat{S}_{k}\#\left( S1 \right) \end{aligned}$$

where $i$ represents the electric field direction and $T_{ijk}=\frac{\partial g_{jk}}{\partial E_{i}}$. Since $g_{jk}=g_{kj}$, $T_{ijk}$ has only 18 independent elements and can be written as

$$\begin{aligned} T_{ijk}=\left[ \begin{matrix} \begin{matrix} T_{xxx} & T_{xyy} & T_{xzz} \\ T_{yxx} & T_{yyy} & T_{yzz} \\ T_{zxx} & T_{zyy} & T_{zzz} \end{matrix} & \begin{matrix} T_{xyz} & T_{xxz} & T_{xxy} \\ T_{yyz} & T_{yxz} & T_{yxy} \\ T_{zyz} & T_{zxz} & T_{zxy} \end{matrix} \end{matrix}\text{ } \right]\#\left( S2 \right) \end{aligned}$$

Further, due to the local symmetry *D*_2_, only $T_{xyz}$, $T_{yxz}$ and $T_{zxy}$ survive.

For an S = 1/2 system, the external static magnetic field ***B_0_*** can be decomposed as $B_{x}=lB_{0}$, $B_{y}=mB_{0}$, $B_{z}=nB_{0}$, where $l=\sin\theta\cos\phi$, $m=\sin\theta\sin\phi$, $n=\cos\theta$. In the rotation experiment, the square of effect g-factor can be calculated by

$$g^{2}=\left[ \begin{matrix} l & m & n \end{matrix} \right]\left[ \begin{matrix} G_{xx} & G_{xy} & G_{xz} \\ G_{yx} & G_{yy} & G_{yz} \\ G_{zx} & G_{zy} & G_{zz} \end{matrix} \right]\left[ \begin{matrix} l \\ m \\ n \end{matrix} \right]\#\left( S3 \right)$$

where $G=g\cdot g^{T}$ is a symmetric matrix. Note that the square of effect g-factor $g^{2}\neq g.g$ as a scalar.

The change of the square of effect g-factor, i.e., $\delta\left( g^{2} \right)=\sum_{i} \frac{\partial g^{2}}{\partial E_{i}}$, is expressed as

$$\begin{aligned} \delta\left( g^{2} \right)=E_{x}\left( B_{xxx}l^{2}+B_{xyy}m^{2}+B_{xzz}n^{2}+{2B}_{xyz}mn+{2B}_{xxz}ln+{2B}_{xxy}lm \right)\# \end{aligned}$$

$$\begin{aligned} +E_{y}\left( B_{yxx}l^{2}+B_{yyy}m^{2}+B_{yzz}n^{2}+{2B}_{yyz}mn+{2B}_{yxz}ln+{2B}_{yxy}lm \right)\# \end{aligned}$$

$$\begin{aligned} +E_{z}\left( B_{zxx}l^{2}+B_{zyy}m^{2}+B_{zzz}n^{2}+{2B}_{zyz}mn+{2B}_{zxz}ln+{2B}_{zxy}lm \right)\#\left( S4 \right) \end{aligned}$$

where $B_{ijk}=\frac{\partial G_{jk}}{\partial E_{i}}$. For proper principal axes, $g_{jk}$ has only 3 non-vanishing elements $g_{xx}$, $g_{yy}$ and $g_{zz}$. Accordingly, the survived $B_{ijk}$ can be simply related to $T_{ijk}$ as

$$\begin{aligned} B_{xyz}=\left( g_{yy}+g_{zz} \right)T_{xyz}\#\left( S5.1 \right) \end{aligned}$$

$$\begin{aligned} B_{yxz}=\left( g_{xx}+g_{zz} \right)T_{yxz}\#\left( S5.2 \right) \end{aligned}$$

$$\begin{aligned} B_{zxy}=\left( g_{xx}+g_{yy} \right)T_{zxy}\#\left( S5.3 \right) \end{aligned}$$

Considering the electric field direction $[111]$ and the rotation axis $[1\bar{2}0]$ of the single crystal coordinate, the directions $(l,m,n)$ of the six groups of Ce^3+^ ions are shown in Table S2-1, where α is the rotation angle during the experiments.

**Table S2-1** direction cosine values for each Ce^3+^ according to the rotation axis $[1\bar{2}0]$.

|  | ***l*** | ***m*** | ***n*** |
| --- | --- | --- | --- |
| Ce-1 | $\sqrt{6}/3\cos\alpha-1/2\sin\alpha$ | $-1/2\sin\alpha$ | $\sqrt{3}/3\cos\alpha+\sqrt{2}/2\sin\alpha$ |
| Ce-2 | $\sqrt{6}/3\cos\alpha+1/2\sin\alpha$ | $1/2\sin\alpha$ | $\sqrt{3}/3\cos\alpha-\sqrt{2}/2\sin\alpha$ |
| Ce-3 | $-\sin\alpha$ | $\sqrt{6}/3\cos\alpha$ | $\sqrt{3}/3\cos\alpha$ |
| Ce-4 | $-1/2\sin\alpha$ | $\sqrt{6}/3\cos\alpha-1/2\sin\alpha$ | $\sqrt{3}/3\cos\alpha+\sqrt{2}/2\sin\alpha$ |
| Ce-5 | $1/2\sin\alpha$ | $\sqrt{6}/3\cos\alpha+1/2\sin\alpha$ | $\sqrt{3}/3\cos\alpha-\sqrt{2}/2\sin\alpha$ |
| Ce-6 | $\sqrt{6}/3\cos\alpha$ | $-\sin\alpha$ | $\sqrt{3}/3\cos\alpha$ |

Combine Table S1-3 and Table S2-1, $\delta\left( g^{2} \right)$ of each Ce^3+^ with applied electric field strength *E* can be calculated as

$$\begin{aligned} \delta\left( g^{2} \right)_{1}={E\sin\alpha\left[ -2\sqrt{6}\left( B_{xyz}+B_{zxy} \right)\cos\alpha+3\left( -2B_{xyz}+B_{zxy} \right)\sin\alpha\right]}/{6\sqrt{3}}\#\left( S6.1 \right) \end{aligned}$$

$$\begin{aligned} \delta\left( g^{2} \right)_{2}={E\sin\alpha\left[ +2\sqrt{6}\left( B_{xyz}+B_{zxy} \right)\cos\alpha+3\left( -2B_{xyz}+B_{zxy} \right)\sin\alpha\right]}/{6\sqrt{3}}\#\left( S6.2 \right) \end{aligned}$$

$$\begin{aligned} \delta\left( g^{2} \right)_{3}=-E\frac{\sqrt{2}}{3}\left( B_{yxz}+B_{zxy} \right)\sin2\alpha\#\left( S6.3 \right) \end{aligned}$$

$$\begin{aligned} \delta\left( g^{2} \right)_{4}=E{\sin\alpha\left[ -2\sqrt{6}\left( B_{yxz}+B_{zxy} \right)\cos\alpha+3\left( -2B_{yxz}+B_{zxy} \right)\sin\alpha\right]}/{6\sqrt{3}}\#\left( S6.4 \right) \end{aligned}$$

$$\begin{aligned} \delta\left( g^{2} \right)_{5}=E{\sin\alpha\left[ +2\sqrt{6}\left( B_{yxz}+B_{zxy} \right)\cos\alpha+3\left( -2B_{yxz}+B_{zxy} \right)\sin\alpha\right]}/{6\sqrt{3}}\#\left( S6.5 \right) \end{aligned}$$

$$\begin{aligned} \delta\left( g^{2} \right)_{6}=-E\frac{\sqrt{2}}{3}\left( B_{xyz}+B_{zxy} \right)\sin2\alpha\#\left( S6.6 \right) \end{aligned}$$

With the Stark shift $\Delta\nu_{e}$, one can calculate $\delta\left( g^{2} \right)=2g^{2}\frac{\Delta\nu_{e}}{v_{mw}}$. Then, $B_{ijk}$ are determined by fitting $\delta\left( g^{2} \right)$ versus rotation angle α with **Eq.S6**, and $T_{ijk}$ are obtained by **Eq.S5**. In the present research, we choose four of the six groups of magnetic inequivalent Ce^3+^ ions for the fitting. Eventually, we obtain

$$B_{xyz}=12.02\times{10}^{-8} m/V$$

$$B_{yxz}=40.21\times{10}^{-8} m/V$$

$$B_{zxy}=33.36\times{10}^{-8} m/V$$

and

$$T_{xyz}=3.30\times{10}^{-8} m/V$$

$$T_{yxz}=8.76\times{10}^{-8} m/V$$

$$T_{zxy}=12.13\times{10}^{-8} m/V$$

Ce-6

Ce-4

Ce-3

Ce-1

**Figure S2-2** The single crystal rotation experiments for detecting the Hamiltonian parameters. Four Ce^3+^ ions of the six are chosen and the best fitting result is found using global searching.

### S3 Echo Decay and Recovery

The electric field inhomogeneity exists due to the fabrication limitation and the electric field strength distribution can be described as a Gaussian distribution

$$P\left( E \right)=\frac{1}{\sqrt{2\pi}\sigma}\exp(-\frac{\left( E-E{}_{0} \right)^{2}}{2\sigma^{2}})$$

where the mean $E{}_{0}={10}^{6} V/m$ and the full width at half maximum (FWHM) is around $0.05\times{10}^{6} V/m$ which is defined by standard deviation $\sigma$ by $FWHM=2\sqrt{2\ln2}\sigma$. This inhomogeneity can cause the decay of electron spin echo. The measured spin echo strength *R* can be calculated as

$$R(t)=\int P\left( E \right)r(E,t)dE$$

where $r(E,t)$ is spin echo intensity for the electric field strength $E$ with acting time $t$.

Now we dive into the calculation of $r(E,t)$. First, the Hamiltonian of spin in external static magnetic field ***B_0_*** can be written as

$$\hat{H}_{0}=g_{\text{eff}} \mu_{B}B_{0}\hat{S}_{z}$$

and the electric-spin coupling Hamiltonian in external static electric field ***E*** as

$$\hat{H}_{E}=\sum_{ijk} E_{i}T_{ijk}\mu_{B}B_{j}\hat{S}_{k}$$

For a two-level energy system, the density matrix of an initial pure state can be written as

$$\rho=\left[ \begin{matrix} 1 & 0 \\ 0 & 0 \end{matrix} \right]$$

Corresponding to the pulse sequence in the experiment (Fig. 2a), the evolution operator of the system can be written as

$$U=e^{-i\hat{H}_{0}\tau}.Rot\left( \pi\right).e^{-i\hat{H}_{0}\left( \tau-t \right)}.e^{-i\left( \hat{H}_{0}+\hat{H}_{E} \right)t}.Rot\left( \frac{\pi}{2} \right)$$

where $Rot$ represents the rotation under microwave pulses, $\tau$ is the time span between two microwave pulses, and $t$ is the acting time of the applied electric field. The final density matrix $\rho^{'}$ is therefore noted as

$$\rho^{'}=U\cdot\rho\cdot U^{\dagger}$$

Then, the spin echo intensity is obtained as

$$r(E,t)=Tr\left[ \rho^{'}{\cdot\sigma}_{x} \right]$$

where $\sigma_{x}$ is the Pauli matrix. It is also possible to simulate the case that the electric field pulse crosses the microwave $\pi$ pulse, where the evolution operator is

$$U^{'}=e^{-i\hat{H}_{0}\left( 2\tau-t \right)}.e^{-i\left( \hat{H}_{0}+\hat{H}_{E} \right)\left( t-\tau\right)}.Rot\left( \pi\right).e^{-i\left( \hat{H}_{0}+\hat{H}_{E} \right)\tau}.Rot\left( \pi/2 \right)$$

Finally, the simulation result is plotted in solid blue line in Fig. S3-1, which clearly shows the decay of electron spin echo.


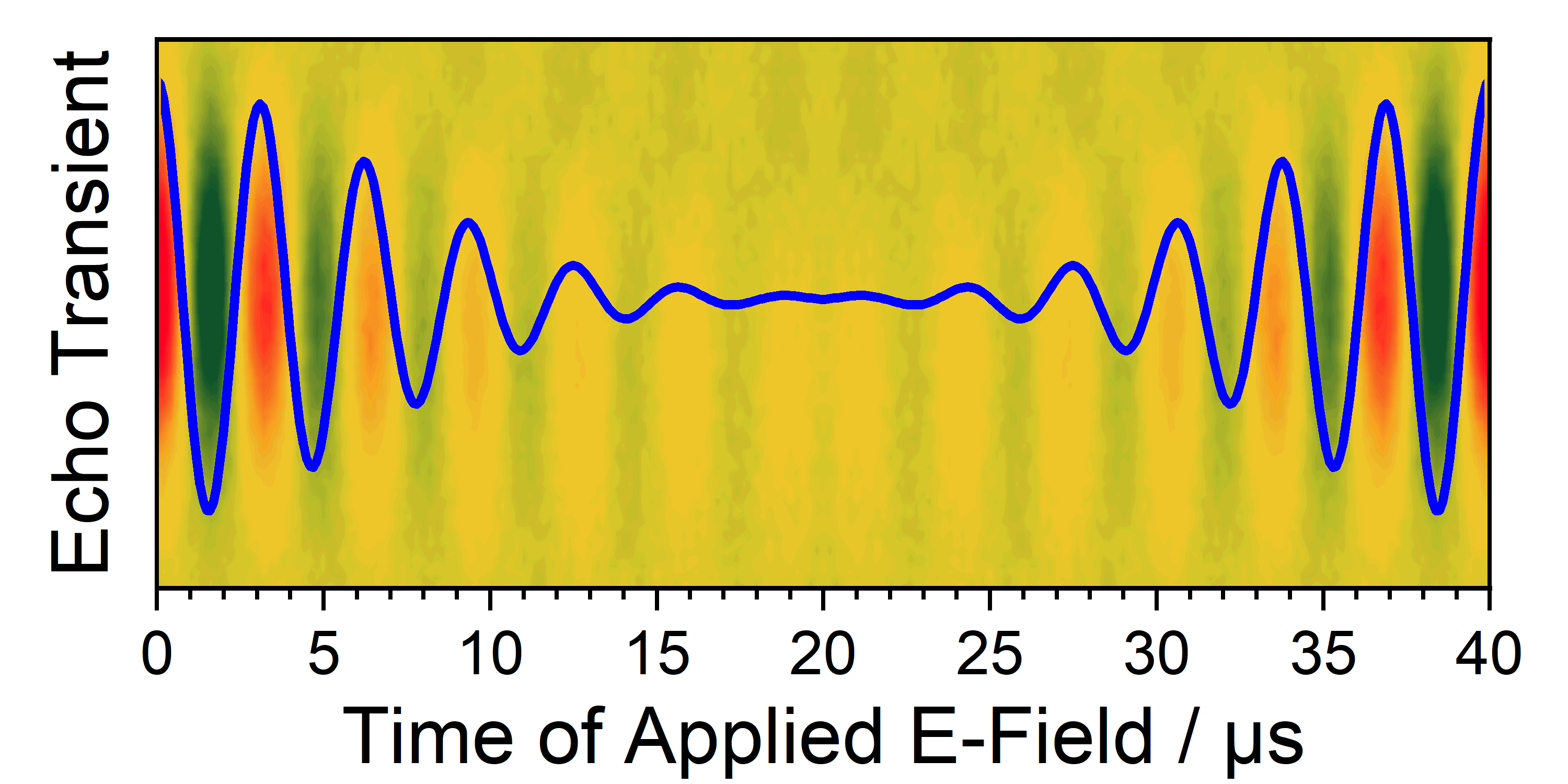


**Figure S3-1** The simulated (blue line) and experimental (background) electron spin echo in the electric field pulse.
